# The Importance of Population Denominators for High-Impact Public Health for Marginalized Populations

**DOI:** 10.2196/publichealth.5883

**Published:** 2016-05-17

**Authors:** David W Purcell, H Irene Hall, Kyle L Bernstein, Thomas L Gift, Eugene McCray, Jonathan Mermin

**Affiliations:** ^1^ Division of HIV/AIDS Prevention National Center for HIV, Viral Hepatitis, STD, and TB Prevention Centers for Disease Control and Prevention Atlanta, GA United States; ^2^ Division of STD Prevention National Center for HIV, Viral Hepatitis, STD, and TB Prevention Centers for Disease Control and Prevention Atlanta, GA United States; ^3^ National Center for HIV/AIDS Viral Hepatitis, STD, and TB Prevention Centers for Disease Control and Prevention Atlanta, GA United States

**Keywords:** HIV surveillance, HIV diagnosis, HIV prevalence, HIV incidence, men who have sex with men, gay and bisexual men, demography

The lack of consistent methods to enumerate population-level denominators for hidden populations has made it difficult for public health to articulate some of the most pressing disparities in America. For example, since the first cases of AIDS in the United States struck gay and bisexual men, injection drug users, and transgender persons, calculating rates of disease to compare impact across populations and geographic areas to highlight disparities and target resources has been challenging. While routine census data have allowed the Centers for Disease Control and Prevention (CDC) to calculate disease rates by sex, age, race/ethnicity, and geographic area [1], the census does not collect information on sexual orientation or same-sex sexual behavior, persons who inject drugs or injection behaviors, heterosexuals who are at higher risk of HIV infection, or transgender persons. This lack of information is nowhere more evident than among gay, bisexual, and other men who have sex with men (MSM), who comprise 67% of estimated number of persons with HIV diagnosed in 2014 (70% when MSM who also inject drugs are included) [1]. Among youth ages 13 to 24, 80% of diagnoses in 2014 were among MSM or MSM who also inject drugs [1]. The impact of HIV on MSM has made them a key focus of the National HIV/AIDS Strategy (NHAS) [2,3]; yet, proportions alone cannot accurately describe disparities, because the size of population denominators vary.

Over the past 5 years, CDC has tried to fill the gap in national, population-wide denominators by using various analytic techniques to estimate the US population size of MSM [4], persons who inject drugs [5], and high-risk heterosexuals [6], and to estimate the population size of MSM and persons who inject drugs by urbanicity and region [7]. Other groups have tried to estimate the size of the population of transgender adults [8] and youth [9]. These national estimates have allowed for the calculation of disease rates for these populations for HIV and other sexually transmitted diseases, which in turn has allowed for national disparities to be highlighted and for federal resources to be better targeted to maximize health impact and increase equity. MSM, who constitute 4% of men in the United States [4], have HIV prevalence and diagnosis rates at least 40 times as great, and syphilis rates at least 60 times as great as for women and other men [4]. However, national estimates may not be applicable to state or local areas because the proportion of the population that is MSM may differ greatly between and within states. Therefore, more refined information is necessary for accurate local information to help plan local programs and allocate resources. 

Currently, the US Census and the American Community Survey (ACS), which provides annual supplemental data to the decennial census data, collect data on same-sex households, which can help enumerate the number of same-sex couples, but cannot, by themselves, lead to a population denominator for MSM nationally or locally. As described by Grey and colleagues [10] in this issue of *JMIR Public Health and Surveillance*, various algorithms have been developed for estimating MSM population size by various geography levels or urbanicity through use of data sources such as national behavioral surveys, census data on same-sex male households, and HIV prevalence among MSM from probability-based samples. Despite these existing methods, there has been an ongoing call from state and local health departments to provide more refined estimates of the size of the MSM population at multiple levels of geography that could be used easily for local purposes and regularly updated with latest data. 

Two CDC divisions worked closely with Grey and colleagues to support development of the refined and updated methods for estimating the size of the MSM population at the state, county, and city levels [10]. This work, which is now available for use by programs, researchers, and other interested parties, took the best of previous methods, recent data, and urbanicity-specific parameters and combined them into an easy-to-use, updatable set of MSM population estimates. This work also introduced a novel imputation approach to estimate MSM in rural areas, where same-sex relationships may be underreported. This approach yielded estimates of MSM population sizes within states, counties, and metropolitan statistical areas (MSAs) in the United States, which provide denominators for calculation of any disease rates, including HIV and STI prevalence and incidence. While we believe that these are the strongest methods available for estimating MSM population size, it is important to acknowledge that these are estimates and are not based on an actual measurement of the population of interest. Methods may continue to improve if more direct data are collected and when stigma related to being part of this partially hidden population decreases so that self-reporting is not potentially hampered by fear.

In itself, this work on MSM denominators represents an important public health tool for public health action [10]. Rosenberg and colleagues [11], also reported in this issue of *JMIR Public Health and Surveillance*, have taken the additional step and applied these denominator estimates to CDC’s HIV surveillance data to provide a snapshot of the devastating impact of HIV on MSM in the United States as a whole, in urban areas nationwide, and in southern states and cities. These data support, at a local level, CDC’s findings using their national population size estimates – that the disproportionate impact of HIV on gay and bisexual men is one of the most extreme disparities amongst the many disparities seen with HIV [4]. The addition of state, city, and county data shows the burden of HIV among MSM in the south.  Most of the cities with the highest rates of MSM living with HIV and new HIV diagnoses are clustered in the south. Six southern states had <15,000 cases and diagnosed prevalence rates of ≥15% [11]. Five highly populated states had ≥15,000 cases and rates between 10% and 15%. Georgia had ≥15,000 cases and ≥15% diagnosed prevalence rate. Of the 25 MSAs with the highest diagnosed prevalence rates in the United States, 21 were in the South and 6 had diagnosed prevalence rates ≥25%. County‐level data showed high rates of diagnosed HIV in both urban and rural counties of the South. These results provide further evidence for the urgent call to action in the National HIV/AIDS Strategy, Updated to 2020, to focus our efforts on MSM, MSM of color, and the southern United States [3].

These important data can be used by local and state health departments to better understand the burden of HIV among MSM and act with the urgency needed to address the disparities between MSM and other population groups, between the south and other regions of the country, and between different regions or cities within states. Since 2010, CDC has pursued a high-impact prevention (HIP) approach to HIV prevention [12]. Through HIP, CDC supports combinations of scientifically proven, cost-effective, and scalable HIV prevention interventions, targeted to the most heavily affected populations and geographic areas. By definition, the South’s disproportionate burden of HIV and disparities makes the region a core focus of prevention efforts. In addition, reducing the impact of HIV in the South is a core focus of NHAS: Updated to 2020 [3]. Since 2010, CDC has greatly increased HIV prevention funding to southern states, by reallocating prevention resources to reflect the burden of the epidemic [13].Closing these gaps is essential to ensuring the health of people in the region and to our nation’s long-term success in ending the epidemic. 

More than ever, we have a broad array of strategies to support high-impact prevention efforts, including condoms, routine and targeted HIV testing, accurate educational and behavioral interventions, antiretroviral therapy to improve the health of people living with HIV and dramatically decrease their likelihood of transmitting HIV, and the use of antiretroviral medicines for pre- or post-exposure prophylaxis [12]. Without a strong focus on MSM, who currently comprise approximately two-thirds of HIV diagnoses [1], we will not be able to meet the ambitious vision of the National HIV/AIDS Strategy to make HIV a rare event in the United States [3,4].

## Abbreviations

ACS: American Community Survey
CDC: Centers for Disease Control and Prevention
HIP: high-impact prevention
HIV: human immunodeficiency virus
MSA: metropolitan statistical area
MSM: men who have sex with men
NHAS: United States National HIV/AIDS Strategy
